# Measuring data reliability for preventive services in electronic medical records

**DOI:** 10.1186/1472-6963-12-116

**Published:** 2012-05-14

**Authors:** Michelle Greiver, Jan Barnsley, Richard H Glazier, Bart J Harvey, Rahim Moineddin

**Affiliations:** 1 Department of Family and Community Medicine, University of Toronto, Duncan Mill Road, Suite 705, Toronto, ON, Canada; 2 Department of Health Policy, Management & Evaluation, University of Toronto, Institute for Clinical Evaluative Sciences, Toronto, Canada; 3 Department of Family and Community Medicine, Institute for Clinical Evaluative Sciences, Centre for Research on Inner City Health, St. Michael’s Hospital, University of Toronto, Toronto, Canada; 4 Dalla Lana School of Public Health, University of Toronto, Toronto, Canada; 5 Department of Family and Community Medicine, University of Toronto, Institute for Clinical Evaluative Sciences, Dalla Lana School of Public Health, Toronto, Canada

**Keywords:** Measurement, Reliability, Validity, Electronic medical records, Preventive health services, Quality of health care, Primary medical care

## Abstract

**Background:**

Improvements in the quality of health care services are often measured using data present in medical records. Electronic Medical Records (EMRs) contain potentially valuable new sources of health data. However, data quality in EMRs may not be optimal and should be assessed. Data reliability (are the same data elements being measured over time?) is a prerequisite for data validity (are the data accurate?). Our objective was to measure the reliability of data for preventive services in primary care EMRs during the transition to EMR.

**Methods:**

Our data sources were randomly selected eligible patients’ medical records and data obtained from provincial administrative datasets. Eighteen community-based family physicians in Toronto, Ontario that implemented EMRs starting in 2006 participated in this study. We measured the proportion of patients eligible for a service (Pap smear, screening mammogram or influenza vaccination) that received the service. We compared the change in rates of selected preventive services calculated from the medical record audits with the change in administrative datasets.

**Results:**

In the first year of EMR use (2006) services decreased by 8.7% more (95% CI −11.0%– − 6.4%, *p* < 0.0001) when measured through medical record audits as compared with administrative datasets. Services increased by 2.4% more (95% CI 0%–4.9%, *p* = 0.05) in the medical record audits during the second year of EMR use (2007).

**Conclusion:**

There were differences between the change measured through medical record audits and administrative datasets. Problems could include difficulties with organizing new data entry processes as well as continued use of both paper and EMRs. Data extracted from EMRs had limited reliability during the initial phase of EMR implementation. Unreliable data interferes with the ability to measure and improve health care quality

## Background

The quality, accuracy and completeness of the information in medical records is fundamental to good patient care and to quality improvement activities: “you cannot improve what you cannot measure” [1]. The transition from paper-based records to Electronic Medical Records (EMRs) has led to expectations that electronic health care data can and will be used to measure and improve the quality of care provided to patients [2,3].

However, the quality of the data entered in EMRs as part of routine care needs to be assessed; missing or inconsistent data may make the measurement of quality problematic [4]. Many physicians continue to simultaneously use both paper and electronic medical records, or “hybrid” charts, [5] leading to uncertainty as to where data reside. Problems already encountered in both Canadian and international primary care EMR settings include inconsistent or missing diagnostic coding and risk factor designation, “dirty data” (misspelled words, inconsistent word strings, free text strings instead of structured data), missing “metadata” or description of data content (for example, referral to “Dr Smith”, where physician specialty is not listed) and data entered in inconsistent or incorrect database fields [4,6-13].

Data quality factors can be categorized as data completeness (are all the data present?), data reliability (are data recorded in the same way across practices and over time?) and data validity (are the data correct?) [14,15].

In a recent literature review of EMR data use for health outcome research, only 24% of studies had a data validation component, and most studies used paper based records as the gold standard [16]. The least common method of validation was the use of administrative data [16,17]. The majority of data validation studies were not conducted in the primary care setting [16]. There were difficulties with the reference standards used to evaluate EMR data quality; some studies relied on unvalidated standards (surveys, interviews); current “gold standards” such as paper notes may be problematic [14,18,19]. As one editorial noted, “there are no agreed reference standards for reporting data quality in primary care and this limits measurement of data quality in electronic patient records” [20]. While there are many possible ways to measure EMR data quality and many areas that can be measured, [8,21,22] systematic reviews of data quality assessment have noted a focus on diagnostic data, laboratory testing, risk factors and demographic information, with limited information on data quality regarding preventive services [7,15].

We recently studied the effects of the first two years of EMR implementation in the practices of eighteen community-based family physicians in Toronto [23]. We measured preventive services targeted by Ontario’s pay for performance program. The pay-for-performance targets were based upon the percentage of a physician’s eligible enrolled patients being provided with Pap smears, mammograms, influenza vaccinations, fecal occult blood screening and primary vaccinations of children under two within a specified timeframe [24-26]. We found no difference in the change of service provision between physicians implementing EMR and a group who continued to use paper records [23].

As part of the study, we evaluated aspects of the quality of the data present for those preventive services in the medical records by comparing results obtained from medical record audits against external data sources. The external sources were provincial administrative claims-based data housed at the Institute for Clinical Evaluative Sciences (ICES). ICES is an independent, not-for-profit health services research organization funded by the Ministry of Health and Long Term Care of Ontario. Ontario has population-based coverage for eligible physician, laboratory, diagnostic imaging and hospital services through the Ontario Health Insurance Plan. A copy of fees submitted to the Ministry of Health and Long Term Care of Ontario is maintained in anonymized administrative databases at ICES.

As noted above, medical record reviews and audits have traditionally been considered to be the “gold standard” for certain health care services, and have been used to validate administrative data [27,28]. The administrative data we used to determine the rates of preventive services have not been validated using this method. However, we assumed that these administrative data captured a relatively constant proportion of services provided over the time during the period we studied. For example, if 75% of influenza vaccinations were available in administrative data in 2006, we would expect that approximately 75% would be available in 2007. We know of no positive or negative changes during the time period studied that would have affected billing proportions captured in administrative databases. We therefore used the change in preventive services reported in the administrative data for the study practices as the reference standard and compared this change to results from the chart audits. This gave us a method of measuring the reliability of data recorded in EMRs over time as compared with administrative data.

Our null hypothesis was that data for preventive services are reliably entered in clinical records over time during EMR implementation; there are no statistically significant or clinically important differences between changes in EMR data and changes in administrative data.

The research question was: does the change in preventive services in medical records before and during the first two years of EMR use agree with the change in preventive services in administrative data?

## Methods

### Participants

The eighteen physicians in this study had previously participated in a pay-for-performance study [29] and data on their characteristics and performance for 2004 and 2005 were available. They changed to a blended capitation model at the end of 2004, in which patients formally register (or roster) with a family physician. These physicians were exposed to pay-for-performance for preventive services in 2005 and began EMR implementation early in 2006. All participating physicians used the same EMR software (Nightingale On Demand®). We studied the change in preventive services in the two years prior to EMR implementation (2004 and 2005) and the first two years of EMR implementation (2006 and 2007).

The principal investigator was also a participant in this study.

### Outcome measures

The study end point was whether or not a preventive service was documented within the required time period for an eligible patient. The target patient population consisted of all eligible enrolled patients. Documentation that the patient received the service through another health care provider was acceptable. Information on services and exclusion criteria is presented in Table 1; children’s vaccinations were not examined, as billing codes in administrative databases also include vaccinations other than the five used in the study.

**Table 1:** Eligibility criteria, exclusion criteria, and required period for preventive service provision

**Service**	**Eligible population**	**Exclusion criteria**	Required period for service provision^*^
Pap smears	Enrolled women age 35 to 69	Previous hysterectomy	Documented service within the past 30 months prior to March 31st
Screening mammograms	Enrolled women age 50 to 69	History of breast cancer	Documented service within the past 30 months prior to March 31st
Influenza vaccination	Enrolled patients age 65 or over		Documented service from October 1st to December 31st of previous year
Fecal occult blood test	Enrolled patients age 50 to 74	History of colorectal cancer; history of inflammatory bowel disease; colonoscopy within the past five years	Documented service in the past 30 months prior to March 31st

^*^The fiscal year end in Ontario’s health care system is March 31st. For example, March 31st 2007 would be considered the 2006 year end, and March 31st 2008 would be considered the 2007 year end.

The denominators were the number of patients eligible for each service who were rostered to the physicians in each cohort by March 31st of each fiscal year (for example, March 31st 2005 for the 2004 fiscal year). Physicians report the preventive service performance levels they have achieved to the Ministry of Health and Long Term Care on March 31st.

The numerators were the number of eligible rostered patients who received a service in the 30 months prior to March 31st of each year for Pap smears, mammograms or fecal occult blood testing, or an influenza vaccination from October 1st to December 31st of the prior year. The rate of service was defined as the proportion of eligible patients receiving a service at least once in the previous 30 months (Pap smears, mammograms, fecal occult blood tests) or in the previous fall (October 1st to December 31st) for influenza vaccination.

We calculated a composite process score [30]. This is calculated by using the total number of medical records audited for eligible patients for each physician as the denominator, and the total number of services recorded in the audits as the numerator. We did not obtain fecal occult blood testing for 2004 as part of our previous study on pay for performance; fecal occult blood testing was not part of the Ontario pay for performance program until 2006. This measure was not included in the composite score due to lack of complete data. The composite process score therefore included mammography, Pap smears and influenza vaccinations from 2004 to 2007.

### Data sources

#### *Chart audits*

We primarily audited the electronic medical records. However, when data were unavailable in an EMR, we retrieved data from the paper chart. We determined that 40 charts per service per provider would be required to achieve a study power of 80% to detect a clinically important increase in service provision of 5% or higher, with an alpha level of .05. To further enhance statistical power, we audited 50 charts per year, per service, per physician.

Five data auditors abstracted data. The research coordinator initially audited ten charts for each service in two practices and reviewed this with the principal investigator. The coordinator then trained each data auditor, and reviewed at least ten charts for each service. The data were independently entered in an Epi Info database [31] by two data entry clerks. Each clerk entered a training sample of at least ten charts for each service. A randomly selected 10% sample of data for each service, each year, and each physician was re-audited and entered in the database; we used the Kappa statistic to compare the two audits.

#### *Administrative audits*

We obtained administrative data for the entire practices from ICES, using the following datasets:

· The ICES Physician Database (IPDB) for information on physician country of graduation;

· The Corporate Provider Database (CPDB) for information on physicians’ Ontario Health Insurance Plan billing number;

· The Ontario’s Registered Persons Database (RPDB) for patient age (as of August 31st 2007), gender and immigration recency by date of OHIP registration [32];

· The Client Agency Program Enrolment (CAPE) tables for information on patient enrolment in each physician’s roster;

· Statistics Canada data on neighborhood income, linked to patients’ residential postal code for estimates of income quintiles [32];

· The Canadian Institute for Health Information’s Discharge Abstract Database for hospital discharge diagnoses;

· The Ontario Health Insurance Plan for billing and diagnostic data to identify patient visits and diagnoses;

· The Ontario Diabetes Database (ODD) for diabetics [28];

· The Ontario Asthma Database (OASIS) [33];

· The Ontario Congestive Heart Failure Database [34];

· The Ontario Chronic Obstructive Pulmonary Disease Database [35];

· The Ontario Hypertension Database [27];

· The Ontario Myocardial Infarction Database [36];

· The Ontario Cancer registry for information on breast or colorectal cancer

· The Ontario Breast Screening Program (OBSP) for information on mammography

A detailed description of the billing codes for the administrative data is provided in Additional file 1.

### Analysis

We first calculated the composite process score for each year, for both medical record audits and administrative data. An equal number of charts had been randomly audited for each service in the medical record; we therefore assigned an equal weight to each service in the administrative dataset (which contained data for the entire practice) for the composite score calculation. We then adjusted for differences in patient age [37] using logistic regression. We used the Generalized Estimating Equation to adjust for the clustering structure of the data in regression models.

Next, we compared the composite process score found in medical record audits and administrative data for each year using the chi-square test. We then compared the year over year change in the composite process score (that is, the percent of change found in the medical record audits and the percent of change found in the administrative data) using logistic regression.

A difference of 5% in the change of services between medical record audits and administrative data was considered clinically important.

Analyses were performed with the use of SAS software, version 9.2 (SAS Institute). All tests were two sided and p values less than 0.05 were considered statistically significant.

The study was approved by the University of Toronto’s Research Ethics Board; the Sunnybrook Research Ethics Board approved the use of ICES data. All physicians provided written informed consent.

## Results

Physician characteristics and patient characteristics are presented in Table 2 and 3, respectively.

**Table 2:** Characteristics of physicians

**Variable**		**Physicians (N = 18)**
Year of graduation*	Median (range)	1977(1964–1992)
Gender*	Male (%)	10(56)
CCFP*	N (%)	11(61)
Number of MDs in practice*	Median (range)	3(1 to 6)
Number of hours worked per week*	Median (range)	42(30 to 60)
Number of patients per physician*	Median (range)	1206(630–2200)
Canadian vs foreign graduate†		16/18

Note: *CCFP* Certificant of the College of Family Physicians of Canada.

*Obtained from self reports.

†Obtained from administrative databases.

**Table 3:** Characteristics of the total practice population

Variable^*^		
Patients	N (Mean)	23,514 (1,306)
Age as of August 31st 2007	Median (IQR)	45 (27–60)
Patient Gender	Male (%)	10,106 (43.0)
Neighbourhood income quintile [32]		
Unknown	N (%)	51 (0.2)
1 (lowest)	N (%)	3,084 (13.1)
2	N (%)	3,643 (15.5)
3	N (%)	4,345 (18.5)
4	N (%)	5,091 (21.7)
5 (highest)	N (%)	7,300 (31.0)
Recent immigrant [32]		1,398 (5.9)
Comprehensiveness of care^†^[32,38]	Mean ± SD	0.54 ± 0.35
Overall morbidity (Resource Utilization Bands)^‡^[39,40]	Mean ± SD	2.73 ± 1.02
0	N (%)	1,047 (4.5)
1	N (%)	1,480 (6.3)
2	N (%)	4,778 (20.3)
3	N (%)	12,567 (53.4)
4	N (%)	2,783 (11.8)
5	N (%)	859 (3.7)
Overall comorbidity (Adjusted Diagnosis Groups)^§^[39,40]	Mean ± SD	4.77 ± 3.04
0	N (%)	1,046 (4.4)
1–4	N (%)	11,189 (47.6)
5–9	N (%)	9,502 (40.4)
10+	N (%)	1,777 (7.6)
Diabetes [28]	N (%)	1,934 (8.2)
Congestive heart failure [34]	N (%)	386 (1.6)
Hypertension [27]	N (%)	5,594 (23.8)
Myocardial infarct [36]	N (%)	311 (1.3)
Asthma [33]	N (%)	3,143 (13.4)
Chronic obstructive pulmonary disease [35]	N (%)	1,120 (4.8)
Mental health [41]	N (%)	4,937 (21.0)

Note: *IQR* interquartile range; *SD* standard deviation.

^*^ Obtained from administrative databases.

^†^ Comprehensiveness of care was determined by measuring the percentage of bills for 21 commonly provided services that were provided by the patient’s own family physician.

^‡^ Resource utilization bands indicate morbidity and expected health care system use, from 0 (lowest) to 5 (highest).

^§^ Adjusted diagnosis groups indicate comorbidity, from 0 groups (lowest level of comorbidity) to 10+ groups (highest level).

A comparison of the composite scores obtained from the medical record audits and from administrative data is presented in Table 4. There was a statistically significant greater increase of 4.2% (95% CI 2.0%–6.4%) in the change in services found in medical record audits following the introduction of pay for performance in 2005. However, this did not reach the previously identified clinically important level of 5%. There was a statistically significant and clinically important larger decrease in services in the first year of EMR when these services were measured using medical record audits rather than administrative data. Measured services declined by 8.7% (95% CI −11.0%– –6.4%) more when measured by medical record audits. There was no statistically significant or clinically important difference in change in the following year

**Table 4:** Comparison of medical record audits and administrative data, composite score for mammography, Pap smears and influenza vaccinations

**Year**	Patients receiving service, medical record audits, n/N, % (95% CI)^*^	**Patients receiving service, administrative data, n/N, % (95% CI)**	**Difference between medical record audits and administrative data within year (95% CI)**	**Difference in the change from previous year (95% CI)**
2004	2264/2807,80.7%(79.2%–82.1%)	11074/14096,78.6%(77.9%–79.2%)	+2.1% (0.5%–3.7%)	
2005 (post pay-for- performance)	2385/2809,84.9%(83.6%–86.2%)	11735/14927,78.6%(78.0%–79.3%)	+6.3% (4.8%–7.8%)	4.2% greater increase with chart audits (2.0%–6.4%)
2006 (first year after EMR introduction)	1995/2696,74.1%(72.4%–75.7%)	11692/15291,76.5%(75.8%–77.1%)	−2.4% (−4.2%– −0.6%)	8.7% greater decrease with chart audits (−11.0%– − 6.4%)
2007 (second year after EMR introduction)	2076/2703,77.2%(75.6%–78.8%)	12005/15559,77.2%(76.5%–77.8%)	0 (−1.7%–1.8%)	2.4% greater increase with chart audits(0%–4.9%)

^*^Percentages are adjusted for patient age.

There was a significantly smaller proportion of services found in the medical record audits as compared with the administrative data in the year that the EMR was introduced (2006). There were more services found in the medical record audits for the two years prior to EMR (2004 and 2005), and no difference was found in the following year (2007).

**Figure 1:**
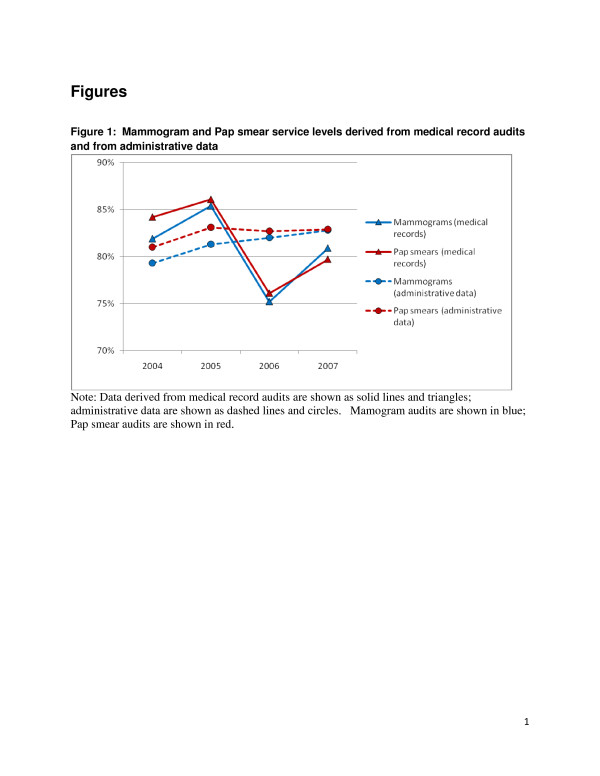
Mammogram and Pap smear service levels derived from medical record audits and from administrative data.

**Figure 2:**
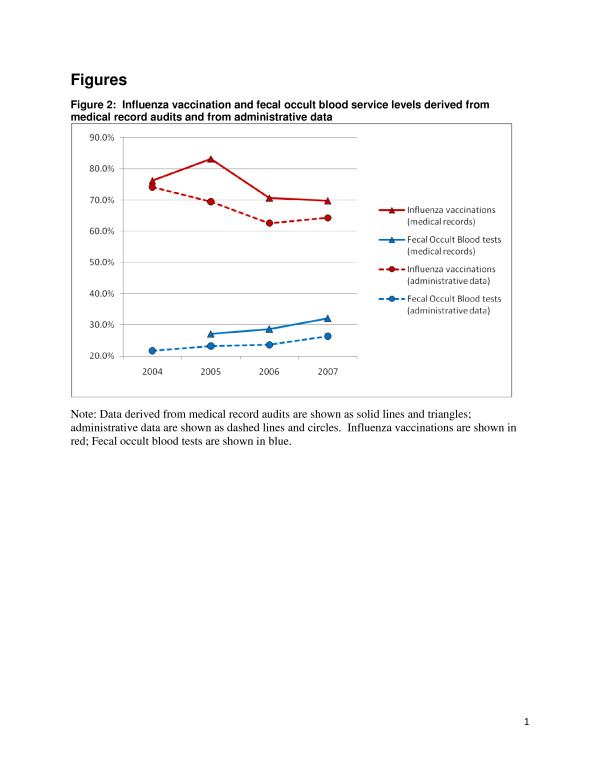
Influenza vaccination and fecal occult blood service levels derived from medical record audits and from administrative data.

Table 5 presents data for individual services derived from medical record audits and administrative data. These are presented graphically in Figure 1 (for Pap smears and mammograms) and Figure 2 (for influenza vaccinations and fecal occult blood tests).

**Table 5:** Individual services levels derived from medical records audits and from administrative data

**Service**	**Source**	**2004**	**2005**	**2006**	**2007**
Fecal occultblood tests,% (n/N)	Administrativedata	21.7% (5139/1117)	23.2% (1216/5239)	23.6% (1232/5216)	26.4% (1355/5137)
Fecal occultblood tests,% (n/N)	Medical records	–	27.1%(236/871)	28.7%(250/870)	32.1%(285/888)
Influenza vaccinations,% (n/N)	Administrativedata	74.2% (3163/4263)	69.5% (3095/4453)	62.6% (2880/4601)	64.3% (3072/4776)
Influenza vaccinations,% (n/N)	Medical records	76.2%(745/978)	83.2%(790/949)	70.7%(638/902)	69.8%(621/901)
Mammograms,% (n/N)	Administrativedata	79.3% (2671/3367)	81.3% (3095/4453)	82.0% (3026/3692)	82.8% (3072/4776)
Mammograms,% (n/N)	Medical records	81.9%(751/917)	85.4%(791/926)	75.2%(672/894)	80.9%(728/900)
Pap smears,% (n/N)	Administrativedata	81.0% (5240/6466)	83.1% (5736/6903)	82.7% (5786/6998)	82.9% (5807/7007)
Pap smears,% (n/N)	Medical records	84.2%(768/912)	86.1%(804/934)	76.1%(685/900)	79.7%(719/902)

The intracluster correlation for each service in medical record audits was generally small, at 0.036 for influenza vaccination, 0.0197 for fecal occult blood testing, 0.0189 for mammography, and 0.009 for Pap smears. Increasing patient age was a significant factor for the provision of a preventive service (data not shown). Using medical record audits, there was a decrease in the provision of mammograms, pap smears and influenza vaccinations between 2005 and 2006 (*p* < 0.001); there was no significant change in fecal occult blood testing (*p* = 0.12).

Administrative data showed no significant change between 2005 and 2006 for Pap smears (*p* = 0.52) or mammography (*p* = 0.48); there was a decrease in influenza vaccination (*p* < 0.0001) and an increase in fecal occult blood testing (*p* < 0.0001).

The overall agreement between the sample of medical records that were re-audited was acceptable (kappa 0.954).

## Discussion

We present a novel method of assessing an aspect of EMR data quality. We compared the rate of change of services over time in the EMR with the rate of change in administrative databases. We found a greater decrease in preventive services in the first year of EMR when data were obtained from medical record audits as opposed to administrative data sources. The discrepancy between the two methods points to the possibility of missing data for preventive services in the medical records.

Reliability reflects data stability, and is necessary (but not sufficient) for data validity [15]. Our administrative data for Pap smears were based on billing codes submitted by laboratories and physician billing codes as detailed in Additional file 1. Laboratory billings would not have been affected by EMR implementation. Administrative data for mammograms were based on radiology billing codes and data from the Ontario Breast Screening Program, as shown in Additional file 1. These also were independent of EMR implementation.

The change in influenza vaccinations was similar in the medical record audits and in the administrative data, perhaps reflecting fewer problems with documentation. Documenting an influenza vaccination does not require looking two years back for the presence of the service, as mammography or Pap smears do; therefore, there may be a less complex workflow associated with recording this service during the move to EMR. There were delays in vaccine delivery in 2006 and 2007, which could account for the lower levels of vaccination found in both the medical record audits and the administrative data during the fall season of those two years.

Challenges with the data needed to measure quality have been reported elsewhere in the literature [7,8,11,14,15]. Roth [42] found that only one-third of the indicators needed for a quality assessment program could be easily extracted from EMRs, and that there were difficulties associated with provider data entry habits and differences across different EMR applications [42]. The structure of the EMR is more complex than that of the paper chart: physicians may not be entering data in consistent or expected locations, making it difficult to extract [42]. Physicians and auditors may have challenges in navigating the electronic medical record. Data from external sources may be scanned in and may not be extractable electronically [43]. Physicians may continue to use both paper and electronic medical records, [5] scattering data across two different systems and possibly increasing the amount of incomplete or duplicated data in audits. Research and quality improvement projects using EMR data will need to consider the quality of data entered in the EMR, as well as issues specific to the EMR application used [42].

Baron described the implementation of a mammography recall program within an innovative, fully computerized primary care group practice [43]. The system was initially unable to properly audit mammograms and to produce accurate lists of patients to be recalled; mammograms were scanned in but were not recognized by the EMR. Baron described the development and implementation of practice processes to “tag” incoming mammograms so that patients could be properly categorized as having or not having had a mammogram within the previous two years [43]. Essentially, the practice cleaned and restructured their mammography data so that data were reliably entered in an area of the EMR where they could be audited.

The quality of information (accuracy, reliability, completeness) has been found to be associated with empirical measures of success in implementing information technology in the business literature [44]. Unreliable information makes a system less useful, impacting implementation efforts and decreasing the net benefits that could be obtained from the technology [45].

Measuring performance depends on accurate documentation [1,46,47]. Once reliable and valid data have been entered into the EMR, interventions that have been found to increase performance, such as audits and feedback to clinicians, [48,49] point of care prompts for needed interventions, [49-51] and reminder letters to patients [52,53] can be effectively implemented. We found a lack of improvement in preventive service documentation associated with the early stages of EMR implementation [23]. It is possible that elements of those negative results were due to problems with data quality during the early EMR implementation efforts.

### Limitations

This study was limited to a group of selected physicians in Toronto. However, all physicians in this study were practicing in community-based settings, similar to the majority of family physicians in Ontario [54]. We studied a single commercially available EMR application, and results may differ for different EMRs. Nonetheless, a recent review of data in a national primary care EMR database using nine different EMR applications found that data quality problems were pervasive across all platforms [4]. Administrative data and patient level data in the EMR could not be linked; we compared practice level data using randomly selected EMR charts. Nonetheless, there is no a priori reason to suspect that there are systematic differences between the two samples.

## Conclusion

In conclusion, we found that, in the early phase of EMR implementation, data for the preventive services we measured were not reliably entered over time in the medical records we audited when compared to provincial administrative data. Data reliability should be assessed if EMR-based data are used to measure and improve quality.

## Competing interests

The authors declare that they have no competing interests.

## Authors’ contributions

All authors contributed to the concept and design of the study. MG and JB contributed to the data gathering. All authors contributed to the analysis, interpretation and preparation of the manuscript. All authors read and approved the final manuscript.

## Pre-publication history

The pre-publication history for this paper can be accessed here:

http://www.biomedcentral.com/1472-6963/12/116/prepub

## Supplementary Material

Additional file 1 Inclusion and exclusion criteria for administrative cohorts. Click here for file
