# Supplementary material for: Measuring data reliability for preventive services in electronic medical records
Source: BMC Health Serv Res. 2012 May 14;12:116. doi: 10.1186/1472-6963-12-116 (PMC3442990; doi:10.1186/1472-6963-12-116)
Supplement: Additional file 1 — Inclusion and exclusion criteria for administrative cohorts. [file 1472-6963-12-116-S1.docx]

**Additional file 1: Inclusion and exclusion criteria for administrative cohorts**

## Pap smears

*Inclusion:* Women 35-69 by January 1st of each year of interest rostered to the physicians (N=18) on March 31, in each of the years of interest (2005-2008 – fiscal 2004-2007). For each woman identified as of March 31st 2005, 2006, 2007 and 2008, their pap smear claims traced back for the past 30 months. Any woman who had at least one of the following tests will be deemed to have been screened:

- Ontario Health Insurance Plan claims G365A, G394A, E430A
- Ontario Health Insurance Plan claims L812, L716, L733

*Exclusion:*

1. Previous diagnosis of cervical cancer (ever)

- ICD-9 180.0, 180.1, 180.8, 180.9

1. Women with hysterectomy (ever)

- OHIP claims S810, S757, S758, S759

3. Died before December 31st 2007

## Mammograms

*Inclusion:* Women 50-69 by January 1st of each year of interest; women rostered to the (N=18) on March 31, in each of the years of interest (2005-2008 – fiscal 2004-2007). For each woman identified as of January 1st 2004, 2005, 2006 and 2007, their mammography claims traced back for the past 30 months. Any woman who had at least one of the following tests will be deemed to have been screened:

- Client screened (SCREENED, from Ontario Breast Screening Program) – where equal to 2 (mammogram only) or 3 (yes, both physical breast exam and mammogram), OR
- Ontario Health Insurance Plan radiology claim X185

*Exclusion:*

1. Breast cancer diagnosed ever

- ICD-9 code: 174
- Data sources: a)CIHI, Same Day Surgery (use discharge dates) or b)Ontario Cancer Registry

2. Died before December 31st 2007

## Influenza vaccinations

*Inclusion:* Persons age 65 or more by January 1st of each year of interest; persons rostered to the physicians (N=18) on March 31, in each of the years of interest (2005-2008 – fiscal 2004-2007). Any person with at least one code for influenza vaccination in the Fall (October 1st to December 31st) of each year of interest (2004, 2005, 2006, 2007) was deemed to be vaccinated:

- Ontario Health Insurance Plan claims G590, G591, G538, G539

*Exclusion:*

- Died before December 31st 2007

## Fecal occult blood testing

*Inclusion:* Persons age 50-74 by January 1st of each year of interest; persons rostered to the physicians (N=18) on March 31, in each of the years of interest (2005-2008 – fiscal 2004-2007). For each person identified as of March 31st 2005, 2006, 2007 and 2008, their fecal occult blood test claims traced back for the past 30 months. Any person who had at least one of the following tests will be deemed to have been screened:

- L181

*Exclusion:*

1. Cases diagnosed with any colorectal cancer between January 1 2000 and December 31st 2007 (using Ontario Cancer Registry).

- ICD-9 codes: 153.0 to 153.4, 153.6 to 154.1

1. Cases diagnosed with any severe inflammatory bowel disease between January 1 2000 and December 31st 2007 (using Canadian Institutes for Health Information, Same Day Surgery and Discharge Abstract Database (use discharge dates))

- ICD-9 codes: 556, 556.0 to 556.9 and 555, 555.0 to 555.9
- ICD-10 codes: K50, K50.0, K50.1, K50.9, K50.9, K51, K51.0-K51.9

1. Cases who have undergone a colonoscopy between January 1 1999 and Dec 31st 2004 (2004 cohort); between Jan 1 2000 and Dec 31 2005 (2005 cohort); between Jan 1 2001 and Dec 31 2006 (2006 cohort); between Jan 1 2002 and Dec 31 2007 (2007 cohort) (according to OHIP)

- Ontario Health Insurance Plan claims Z555 plus one of E740 or E741 or E747 or E705 on the same day

1. Died before December 31, 2007
